# Postoperative complications in adult patients undergoing surgery with confirmed infection by SARS-CoV-2: An integrative review

**DOI:** 10.1590/1518-8345.5346.3496

**Published:** 2021-11-08

**Authors:** Erica Favaro, Daiane Rubinato Fernandes, Leticia Genova Vieira, Amanda Salles Margatho, Karina Dal Sasso Mendes, Renata Cristina de Campos Pereira Silveira

**Affiliations:** 1 Universidade de São Paulo, Escola de Enfermagem de Ribeirão Preto, PAHO/WHO Collaborating Centre for Nursing Research Development, Ribeirão Preto, SP, Brazil.; 2 Scholarship holder at the Coordenação de Aperfeiçoamento de Pessoal de Nível Superior - (CAPES), Brazil.; 3 Scholarship holder at the Conselho Nacional de Desenvolvimento Científico e Tecnológico/Ministério da Ciência, Tecnologia e Inovações, Brazil.

**Keywords:** Postoperative Complications, Coronavirus Infections, SARS-CoV-2, Adult, Perioperative Nursing, Review Literature as Topic, Complicaciones Posoperatorias, Infecciones por Coronavirus, SARS-CoV-2, Adulto, Enfermería Perioperatoria, Literatura de Revisión como Asunto, Complicações Pós-Operatórias, Infecções por Coronavírus, SARS-CoV-2, Adulto, Enfermagem Perioperatória, Literatura de Revisão como Assunto

## Abstract

**Objective:**

to analyze the evidence available in the literature about postoperative complications in adult patients undergoing surgical procedures with confirmed infection by SARS-CoV-2.

**Method:**

an integrative literature review conducted in the CINAHL, EMBASE, LILACS, PubMed, Scopus and Web of Science databases, as well as in the gray literature. The references identified were exported to the EndNote manager and, subsequently, to the Rayyan web application for study selection. The stages of sampling, categorization of studies, evaluation of the studies included, interpretation of the results and knowledge synthesis were performed by two reviewers independently and in a masked manner. The data were analyzed descriptively.

**Results:**

of the 247 articles identified, 15 were selected to comprise this review. The prevalent postoperative complications in patients infected with SARS-CoV-2 were the following: cough, dyspnea and hypoxia, need for invasive mechanical ventilation or not, admission to the intensive care unit and death.

**Conclusion:**

the most reported postoperative complications in the studies evaluated were respiratory-related, followed by cardiovascular complications. The importance of preoperative screening for COVID-19 is highlighted, as well as of the monitoring and tracking of confirmed cases in the postoperative period, as these actions exert an impact on reducing the occurrence of complications related to SARS-CoV-2.

## Abstract (es)

**Objetivo:**

analizar la evidencia disponible en la literatura sobre las complicaciones postoperatorias en pacientes adultos sometidos a procedimientos quirúrgicos con infección confirmada por SARS-CoV-2.

**Método:**

revisión integradora de la literatura realizada en las bases de datos CINAHL, EMBASE, LILACS, PubMed, Scopus, *Web of Science* y literatura gris. Las referencias identificadas se exportaron al administrador *EndNote* y luego a la aplicación *web* Rayyan para la selección de estudios. Las etapas de muestreo, categorización de los estudios, evaluación de los estudios incluidos, interpretación de los resultados y síntesis del conocimiento fueron realizadas por dos revisores de forma independiente y enmascarada. Los datos se analizaron de forma descriptiva.

**Resultados:**

de los 247 artículos identificados, se seleccionaron 15 para componer esta revisión. Las complicaciones postoperatorias prevalentes en pacientes infectados por SARS-CoV-2 fueron: tos, disnea e hipoxia, necesidad de ventilación mecánica invasiva o no, ingreso en la unidad de terapia intensiva y muerte.

**Conclusión:**

las complicaciones postoperatorias más reportadas en los estudios evaluados fueron las respiratorias seguidas de las cardiovasculares. Se destaca la importancia del cribado preoperatorio de COVID-19, así como también del monitoreo y seguimiento de los casos confirmados en el postoperatorio, ya que son acciones que reducen la aparición de complicaciones relacionadas con el SARS-CoV-2.

## Abstract (pt)

**Objetivo:**

analisar as evidências disponíveis na literatura sobre as complicações pós-operatórias em pacientes adultos submetidos a procedimentos cirúrgicos com infecção confirmada por SARS-CoV-2.

**Método:**

revisão integrativa da literatura realizada nas bases de dados CINAHL, EMBASE, LILACS, PubMed, Scopus, *Web of Science* e na literatura cinzenta. As referências identificadas foram exportadas para o gerenciador EndNote e, em seguida, para o aplicativo web Rayyan para a seleção dos estudos. As etapas de amostragem, categorização dos estudos, avaliação dos estudos incluídos, interpretação dos resultados e síntese do conhecimento foram realizadas por dois revisores de forma independente e mascarada. Os dados foram analisados de forma descritiva.

**Resultados:**

dos 247 artigos identificados, 15 foram selecionados para compor a presente revisão. As complicações pós-operatórias prevalentes em pacientes infectados com SARS-CoV-2 foram: tosse, dispneia e hipóxia, necessidade de ventilação mecânica invasiva ou não, internação em unidade de terapia intensiva e óbito.

**Conclusão:**

destacaram-se, como complicações pós-operatórias mais relatadas nos estudos avaliados, as respiratórias seguidas das cardiovasculares. Ressalta-se a importância da triagem pré-operatória para COVID-19, bem como de acompanhamento e rastreamento de casos confirmados no pós-operatório, por serem ações que impactam na redução da ocorrência de complicações relacionadas ao SARS-CoV-2.

## Introduction

The Coronavirus 2019 disease (COVID-19) was first identified in Wuhan, province of Hubei, China, in December 2019, and quickly spread around the world. In March 2020, it was declared a pandemic by the World Health Organization (WHO). It is an infectious disease caused by the etiological agent called Coronavirus 2 of Severe Acute Respiratory Syndrome (*Coronavirus 2 - SARS-CoV-2*)^(1)^.

The clinical manifestations caused by COVID-19 are usually related to the upper respiratory tract, with the majority of those infected being asymptomatic or with mild symptoms. The most frequent clinical signs and symptoms are fever, dry cough, myalgia or fatigue and dyspnea and, less frequently, headache, diarrhea, nausea, vomiting, anosmia, dysgeusia and sore throat. Some patients can develop lower respiratory tract infections. However, infections can progress to pneumonia with Severe Acute Respiratory Syndrome (SARS), renal failure, multiple organ dysfunction syndrome and death^(2-4)^.

Given the high rates of infection and transmissibility, there was a significant increase in patients with acute diseases, which overloaded health systems around the world, especially hospitals, which were not prepared to deal with the magnitude of care and resources required by this pandemic. Overcrowding of the Intensive Care Units and overload of health professionals were inevitable, requiring the rapid adaptation of the surgical sectors^(5-9)^.

To relieve pressure on the health system and minimize the risk of nosocomial spread of COVID-19 during surgical procedures, specialized societies determined that elective surgical interventions be suspended or postponed. However, urgent and emergency procedures, as well as those with urgent surgical indication, should undergo a careful assessment on a case-by-case basis, to analyze the risk of transmission and postoperative complications^(9-10)^. In addition to that, measures to prevent and avoid the spread of the virus within the surgical environment highlighted the correct use of personal protective equipment and the reduction in the transit of personnel within operating rooms during invasive procedures^(2,8)^.

Studies evaluating the impact of postoperative complications in patients infected with SARS-CoV-2 are still scarce in the literature. Despite this, it was evidenced that, due to proinflammatory cytokine and immunosuppressive responses related to surgery and mechanical ventilation, such patients are especially susceptible to subsequent pulmonary complications, changes in laboratory tests, acute kidney injury, arrhythmia, acute cardiac injury, shock and secondary infections^(11-14)^.

Identifying postoperative complications early is relevant to reduce morbidity and mortality in this period. A number of studies show that previously undiagnosed COVID-19 can complicate postoperative recovery^(13,15)^. In this sense, the importance of the Nursing team in the post-anesthetic recovery room and in the inpatient units is highlighted, as this is the professional category that stays the longest with the patient, being responsible for evaluating complications and implementing interventions aimed at preventing problems and promoting health recovery^(13,15)^.

Despite the publication of guidelines to guide the ways of reorganizing surgical activities during the COVID-19 pandemic, the scientific production related to the management of surgeries is incipient, particularly with regard to the treatment of postoperative complications. Added to this context, the nurse is the protagonist to early identify the clinical manifestations that may be related to such adversities and make evidence-based clinical decisions to solve them. Thus, synthesizing the evidence to increase the safety of surgical patients exposed to SARS-CoV-2 is urgent and necessary, with the potential to directly influence the clinical outcomes of these patients. For this reason, this study aimed at analyzing the evidence available in the literature on postoperative complications in adult patients undergoing surgical procedures with confirmed infection by SARS-CoV-2.

## Method

### Study type

**Figure 1:**
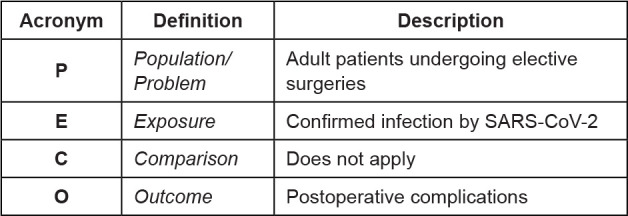
PECO strategy to formulate the research question. Ribeirão Preto, SP, Brazil, 2020

This study consists of an integrative literature review, filed on the Open Science Framework platform, whose registration is available at https://osf.io/be97s/ which enables to gather and synthesize the production of knowledge on a given subject matter, ensuring, through the wide number of studies, theoretical deepening on different perspectives of the same theme^(16)^. The study was conducted in six stages^(16)^, namely: identification of the theme, sampling, categorization of the studies, evaluation of the studies included, interpretation of the results and knowledge synthesis, respectively. The question of this integrative review was guided by the PECO strategy^(17)^ (Figure 1) and consisted of: Which are the postoperative complications in adult patients undergoing surgical procedures with confirmed infection by SARS-CoV-2?

### Data collection

**Figure 2:**
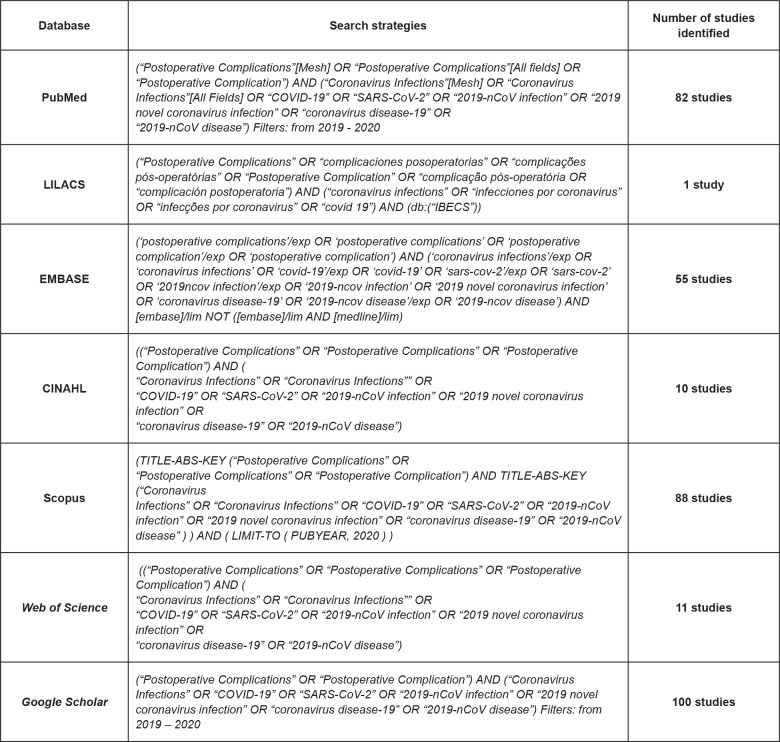
Search strategies used in the databases used. Ribeirão Preto, SP, Brazil, 2020

To identify the studies, the following electronic databases were used: CINAHL, EMBASE, LILACS, PubMed, Scopus and *Web of Science*. The gray literature was consulted using Google Scholar. The search strategy was formulated with the combination of the following controlled descriptors and/or keywords “*Postoperative Complications*”, “*Coronavirus Infections*”, “COVID-19” and their respective synonyms, combined with Boolean operators (AND and OR), and adapted according to the specifics of each database. The search strategy conducted in each database is described in Figure 2.

After the search, the results were exported to the EndNote Basic^(18)^reference manager, online version, to remove duplicate references. Subsequently, they were imported into the Rayyan web application, which can be accessed through https://rayyan.qcri.org for the selection of studies.

In the Rayyan web application, the studies were evaluated and selected by two reviewers independently and blindly, first by reading the titles and abstracts, in order to verify if they met the eligibility criteria of this review. The studies considered eligible were then analyzed by reading the text in its entirety, according to the eligibility criteria. In case of disagreement between the reviewers, a third reviewer with expertise in the subject matter was consulted.

### Period

The search in the electronic databases was carried out on August 19^th^, 2020.

### Selection criteria

Primary studies addressing postoperative complications occurring in adult patients undergoing surgery and infected with COVID-19, published in Portuguese, English or Spanish, were included. Studies conducted with pediatric patients, conference proceedings and abstracts, and studies that did not meet the scope of this review were excluded.

A total of 247 studies were identified in the databases, of which 87 were excluded for being duplicates in at least two databases, totaling 160 studies. Among the 160 studies identified and evaluated by reading titles and abstracts, 96 were excluded for not meeting the eligibility criteria of this review. Of the 64 studies eligible for analysis by reading the text in its entirety, 15 were included in this review. At the end of the selection process, a manual search was carried out in the list of references of the included studies. However, it did not identify publications that could be included in the final review sample. Thus, the final sample consisted of 15 primary studies.

### Instrument used to collect the information

The data from the studies were collected using an adapted form^(19)^, which includes the following: reference and year of publication, country where the study was conducted, methodological characteristics [study design according to the nomenclature used by the author(s) and sample] and main outcomes (postoperative complications).

### Data treatment and analysis

The data were analyzed qualitatively, synthesizing the evidence from the primary studies in a descriptive way.

For the critical evaluation stage, it was decided to assess the methodological quality of the primary studies included in the sample, using the tools provided by the Joanna Briggs Institute (JBI)^(20)^, also independently, by two reviewers. This evaluation was carried out considering the appropriate tools for each type of design included, which can present “yes”, “not clear”, “no” or “not applicable” answers. Before initiation of the critical evaluation of the studies, decisions about the scores were agreed upon between the reviewers. The studies included were categorized for risk of bias as follows: high risk of bias (when it reached a “yes” score below 49%), moderate risk of bias (when the “yes” score reached 50% to 69%), and low risk of bias (when the study reached a “yes” score above 70%)^(21)^. The third reviewer was consulted in case of conflicts in the assessment between the first two reviewers.

Considering that it is fundamental to unite methodological quality and the strength of the evidence for decision-making in the clinical practice, the evaluated studies were classified according to the level of evidence, according to the hierarchy for clinical issues of prognosis/prediction or etiology, which varies from level I (evidence from synthesis of cohort studies or case-control studies) to level V (evidence from experts’ opinion)^(22-23)^.

## Results

**Figure 3:**
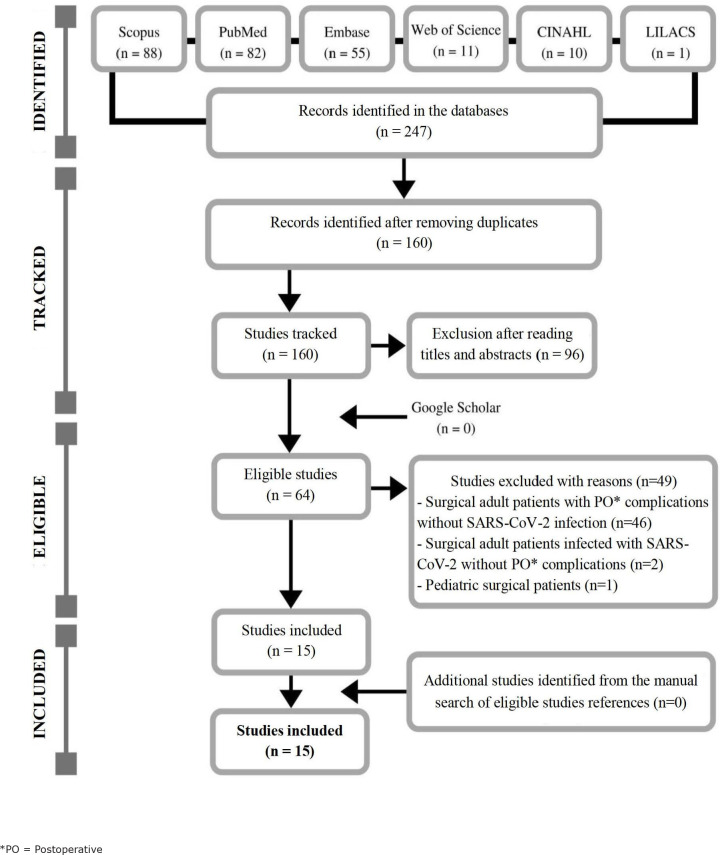
Adaptation of the study selection flowchart of this integrative review (n=15), according to the Preferred Reporting Items for Systematic Reviews and Meta-Analyses (PRISMA) model^(37)^. Ribeirão Preto, SP, Brazil, 2020

This integrative review analyzed 15 primary studies that identified postoperative complications in adult patients undergoing surgeries with confirmed SARS-CoV-2 infection, which were published in 2020, in English (n=14) and Spanish (n =1), in international journals. The flowchart corresponding to the selection of studies can be seen in Figure 3.

The analysis allowed identifying four articles characterized as cohort studies^(9,24-26)^, three cross-sectional studies^(27-29)^, three case reports^(30-32)^, two retrospective studies^(15,33)^, two case series^(34-35)^ and a letter to the editor^(36)^.

**Figure 4:**
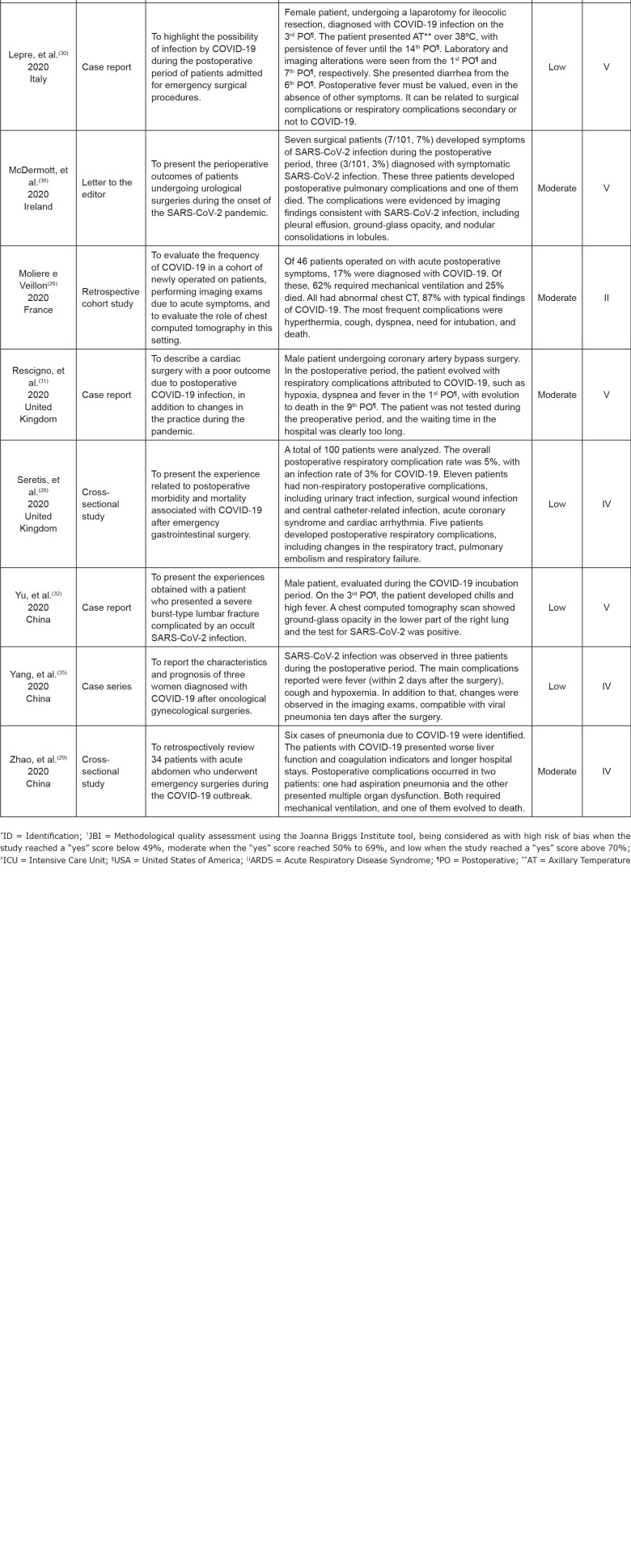
Synthesis chart of the studies included to compose the final sample of this integrative review (n=15). Ribeirão Preto, SP, Brazil, 2020

Figure 4 presents the general synthesis of the studies included in this review by author, year of publication, country, method, objective, main results (herein represented by the postoperative complications in adult patients infected with SARS-CoV-2), methodological quality and level of evidence.

The main postoperative complications identified in the primary studies were related to the respiratory system, the most prevalent being cough^(15,25-26,34-35)^, dyspnea^(26,31,34)^, hypoxia^(25,31,34-35)^, severe respiratory failure^(24,28,33)^, pulmonary embolism^(9,28-29)^ and severe respiratory infection requiring invasive or non-invasive mechanical ventilation^(9,25-26,34)^.

Patients with confirmed infection by SARS-CoV-2 presented changes in their imaging exams, compatible with pneumonia caused by COVID-19, such as ground-glass opacity, nodular consolidations in lobules and pleural effusion^(25-26,30,32,35-36)^. Changes in laboratory tests evidenced metabolic acidosis^(24)^, coagulation disorders^(24,29-30)^ and acute kidney injury^(24)^.

A number of studies emphasize the importance of preoperative screening for COVID-19 for all patients, in order to exclude the possibility of infection, considering the incubation period of the virus for patients with negative results^(32,35)^. Some authors report difficulty in diagnosing postoperative SARS-CoV-2 infection^(30,36)^, as the symptoms are similar to those of common postoperative complications, such as increased body temperature^(15,25-26,30-32,35)^.

Other complications observed were related to the cardiovascular system, including acute myocardial infarction^(24)^, hypotension^(25)^, acute cardiac injury^(15)^ and cardiac arrhythmia^(15,28)^. In addition to that, cases of septic shock^(24)^, urinary tract infection^(28,34)^ and multiple organ dysfunction were observed^(15,24)^.

The studies also evidenced cases of need for second surgeries^(9)^, unplanned admission to the ICU^(9,15,34)^ and death^(15,25-26,29,33-34,36)^, resulting from worsening of the post-operative complications among patients with confirmed SARS-CoV-2 infection.

Regarding the methodological quality of the primary studies included, eight were classified as with low risk of bias and, therefore, they present good methodological quality. Six studies were classified as with moderate risk of bias and moderate methodological quality, and only one as with high risk of bias and low methodological quality. Regarding the level of evidence, four studies presented level II, seven were level IV and four, level V.

## Discussion

This study synthesized the evidence related to the postoperative complications in adult patients undergoing surgical procedures with SARS-CoV-2 infection and verified that the most reported complications in the primary studies included in this review were related to the respiratory system and associated with high mortality rates among the patients undergoing surgeries. Cough, dyspnea and hypoxia, image changes compatible with the COVID-19 disease, and the need for invasive mechanical ventilation were among the most frequently found in the studies analyzed.

Respiratory complications are common in the postoperative period in general, mainly due to the anesthetic procedure. In general anesthesia, given the need for orotracheal intubation, changes occur in the pulmonary system due to changes in respiratory impulse and muscle function in the anesthetized patient, reducing lung volumes and, in many cases, leading to complications such as atelectasis. The respiratory system can take up to six weeks to return to its baseline condition after general anesthesia for major surgeries^(38)^.

However, the incidence of postoperative respiratory complications during the pandemic is even higher. A multicenter international cohort study, carried out between January and March 2020 in 235 hospitals from 24 countries, with 1,128 patients undergoing surgeries with confirmed infection by SARS-CoV-2, found a 51.2% incidence of pulmonary complications^(9)^. This rate is higher than that identified in a multicenter cohort study, carried out pre-pandemic from 2014 to 2015, in 211 hospitals from 28 European countries with 21,694 adult patients undergoing general anesthesia, in which the incidence of postoperative pulmonary complications was 7.6%^(39)^.

Among the risk factors for the development of respiratory complications in the postoperative period, comorbidities (systemic arterial hypertension, chronic obstructive pulmonary disease and cancer) stand out, as well as extrinsic factors such as smoking and the surgical procedure itself, which can lead to impairment of the immune system cells^(40-41)^. Added to these factors, the infection by the new coronavirus presents itself as an additional risk factor for worsening of the postoperative complications, since SARS-CoV-2 presents tropism for the cells of the respiratory system^(9,42-45)^ and increases the pro-inflammatory cytokines and chemokines levels, correlated with disease severity^(46-47)^.

The signs of SARS-CoV-2 infection in the postoperative period can manifest themselves very similarly to common infections, such as surgical site infections, hindering COVID-19 diagnosis. Therefore, fever episodes in the postoperative period, even if incidental and without the presence of other signs and symptoms, must be carefully investigated, as they can be related to surgical or respiratory complications arising from SARS-CoV-2 or from another microorganism infection^(30,36)^.

When perioperative SARS-CoV-2 infection is identified, the prognosis tends to be worse, with a significant increase in the mortality rates, length of stay and need for mechanical ventilation, either invasive or not. Therefore, preoperative screening is recommended to detect SARS-CoV-2 infection in all patients undergoing elective surgical procedures. However, it is known that this practice is not possible in all surgical services. In addition to that, the virus incubation time and the possibility of perioperative infection must be taken into account during screening, and postoperative testing is also recommended^(24,29,32,35-36)^. For this reason, a number of studies suggest that each case be evaluated individually, in relation to the risks associated with perioperative SARS-CoV-2 infection, when compared to the risks of delaying the performance of surgical procedures. Male patients aged 70 years old or more, with comorbidities, and patients classified as ASA (American Society of Anesthesiologists) from 3 to 5, undergoing oncologic surgeries, major surgeries or emergency surgeries, are the most vulnerable to adverse outcomes^(9,45)^.

This synthesis also evidenced other complications among the surgical patients infected with SARS-CoV-2, mainly cardiovascular complications such as arrhythmia, acute cardiac injury and acute myocardial infarction. Corroborating these results, a recent study showed that SARS-CoV-2 has a pathogenicity that can increase myocardial damage^(48)^. The results of this research showed cases of acute cardiac injury, shock and arrhythmia in 7.2%, 8.7% and 16.7% of the infected patients, respectively, being more prevalent among those who needed intensive care. Based on these data, careful attention must be given to cardiovascular protection during treatment for COVID-19^(49)^, especially in the postoperative period.

The Nursing team plays a leading role in the care provided to surgical patients. Perioperative Nursing care based on scientific evidence is essential to prevent postoperative complications^(50-51)^ and also to prevent and reduce the transmission of SARS-CoV-2 in surgical environments.

The need of new research studies on the subject matter is highlighted, pointing out the effects of the SARS-CoV-2 infection on the prognosis of surgical patients, so that the health team can intervene early and ensure patient safety in the postoperative period.

Among the weaknesses of this study, it is highlighted that most of the articles analyzed addressed problems of a respiratory nature, which can hinder the analysis of other complications experienced by surgical patients with SARS-CoV-2. It is also noteworthy that, of the 15 studies, 11 presented a classification of evidence level between IV and V, and that seven had a vulnerable methodological quality, which can compromise generalization of the results to other contexts. Among the knowledge gaps identified, there was lack of studies that addressed complications of other natures, in addition to the respiratory system. New studies with robust methodological approaches and that comprehensively identify systemic complications are recommended.

## Conclusion

Considering the diverse evidence synthesized on the postoperative complications that affected adult patients infected with SARS-CoV-2 and undergoing surgeries, it is concluded that the main complications are related to the respiratory system, with increased mortality rates, need for hospitalization in intensive care unit and prolonged hospital stay. This occurrence can be explained by the fact that SARS-CoV-2 has greater affinity with respiratory epithelial cells. In addition to that, complications related to the cardiovascular system and other systemic complications were observed in this population.

The importance of rigorous preoperative screening that meets at least the majority, but preferably all patients undergoing surgical procedures, is highlighted, taking into account the incubation period of the virus, monitoring and tracking of confirmed cases in the post-operative period, in order to reduce the occurrence of complications related to the SARS-CoV-2 infection.
